# Effects of metformin and other biguanides on oxidative phosphorylation in mitochondria

**DOI:** 10.1042/BJ20140620

**Published:** 2014-08-22

**Authors:** Hannah R. Bridges, Andrew J. Y. Jones, Michael N. Pollak, Judy Hirst

**Affiliations:** *Medical Research Council Mitochondrial Biology Unit, Wellcome Trust/MRC Building, Hills Road, Cambridge, CB2 0XY, U.K.; †Department of Oncology, McGill University and Lady Davis Research Institute of the Jewish General Hospital, Montreal, Quebec, Canada, H3T 1E2

**Keywords:** ATP synthase, biguanide, complex I, metformin, NADH:quinone oxidoreductase, reactive oxygen species (ROS), AMPK, AMP-activated protein kinase, DMEM, Dulbecco’s modified Eagle’s medium, ECAR, extracellular acidification rate, FeCN, ferricyanide, HAR, hexaammineruthenium(III), OCR, oxygen consumption rate, OCT1, organic cation transporter 1, ROS, reactive oxygen species, SMP, submitochondrial particle

## Abstract

The biguanide metformin is widely prescribed for Type II diabetes and has anti-neoplastic activity in laboratory models. Despite evidence that inhibition of mitochondrial respiratory complex I by metformin is the primary cause of its cell-lineage-specific actions and therapeutic effects, the molecular interaction(s) between metformin and complex I remain uncharacterized. In the present paper, we describe the effects of five pharmacologically relevant biguanides on oxidative phosphorylation in mammalian mitochondria. We report that biguanides inhibit complex I by inhibiting ubiquinone reduction (but not competitively) and, independently, stimulate reactive oxygen species production by the complex I flavin. Biguanides also inhibit mitochondrial ATP synthase, and two of them inhibit only ATP hydrolysis, not synthesis. Thus we identify biguanides as a new class of complex I and ATP synthase inhibitor. By comparing biguanide effects on isolated complex I and cultured cells, we distinguish three anti-diabetic and potentially anti-neoplastic biguanides (metformin, buformin and phenformin) from two anti-malarial biguanides (cycloguanil and proguanil): the former are accumulated into mammalian mitochondria and affect oxidative phosphorylation, whereas the latter are excluded so act only on the parasite. Our mechanistic and pharmacokinetic insights are relevant to understanding and developing the role of biguanides in new and existing therapeutic applications, including cancer, diabetes and malaria.

## Abstract 2

Metformin is a widely prescribed anti-diabetic, but its mode of action is disputed. It has been proposed to inhibit respiratory complex I. We show how metformin and related compounds affect both complex I and ATP synthase in mammalian mitochondria.

## INTRODUCTION

Metformin is the most commonly prescribed drug for Type II diabetes. It decreases blood glucose by mechanisms thought to involve the activation of AMPK (AMP-activated protein kinase) [1] and/or inhibition of adenylate cyclase [2] in response to energetic stress, and/or the direct inhibition of mitochondrial glycerophosphate dehydrogenase [3]. In addition to its anti-hyperglycaemic activity, metformin is under investigation as a potential anti-neoplastic agent [4,5], and has been observed to decrease cardiac ischaemia/reperfusion injury [6]. The primary effect of metformin is generally thought to be the inhibition of respiratory complex I (NADH:ubiquinone oxidoreductase) that leads to energetic stress by decreasing ATP synthesis by oxidative phosphorylation. This consideration stems from two pieces of circumstantial evidence: inhibition of NADH-linked respiration by metformin has been observed in mitochondria, submitochondrial particles [7] and immunoprecipitated complex I [8], and inhibition of complex I can inhibit respiration [9] and lead to activation of AMPK in cells [10]. However, metformin is only a weak inhibitor of complex I, there is no molecular-level or mechanistic knowledge about the metformin–complex I interaction, and metformin has been reported to inhibit several other enzymes also [3,11–13]. Furthermore, many studies have used supraphysiological extracellular or extramitochondrial biguanide concentrations, and there are many alternative and diverse mechanisms for AMPK activation [10]. Therefore causative connections between complex I inhibition by metformin, and the physiological consequences of metformin treatment, remain contentious.

Complex I (NADH:ubiquinone oxidoreductase) [14] is crucial for respiration and oxidative phosphorylation in mammalian mitochondria. It oxidizes NADH in the mitochondrial matrix, produced by the tricarboxylic acid cycle and β-oxidation of fatty acids and delivered from glycolysis in the cytosol by the malate–aspartate shuttle, regenerating the NAD^+^ pool. It uses the two electrons from NADH oxidation to reduce ubiquinone to ubiquinol in the mitochondrial inner membrane, supplying respiratory complexes III and IV with electrons for the reduction of O_2_ to water. The energy released by the NADH:ubiquinone redox reaction is used to transport protons across the inner membrane, supporting the protonmotive force (Δ*p*) that drives ATP synthesis by F_1_F_O_-ATP synthase. Complex I is also an important source of ROS (reactive oxygen species) production in mitochondria, with the rate of production dependent on the redox status of the NADH/NAD^+^ pool in the matrix [15,16].

Metformin is a member of the biguanide family; the biguanide functional group comprises two guanidiniums joined by a common nitrogen. Phenformin and buformin are also biguanides with anti-diabetic properties, but their clinical use has been discontinued due to increased incidence of lactic acidosis [17], a known consequence of complex I inhibition. Biguanides are also used to prevent and treat malaria. The anti-malarial biguanide proguanil and its metabolite cycloguanil exhibit two distinct activities against *Plasmodium:* proguanil acts synergistically with atovaquone to collapse the mitochondrial membrane potential [18], and cycloguanil inhibits *Plasmodium* dihydrofolate reductase [19]. Little is known about the interaction(s) between biguanides and the mitochondrial oxidative phosphorylation complexes, as biguanides do not structurally resemble either the substrates or canonical inhibitors of any of these enzymes. However, it is known that the positive charge on the biguanide moiety results in accumulation of biguanides in the mitochondrial matrix (in response to the plasma and mitochondrial membrane potentials, and subject to transport processes) to concentrations up to 1000-times greater than in the extracellular environment. Consequently, very high concentrations of biguanides are relevant for testing on isolated mitochondrial enzymes and membranes, even though they greatly exceed the low extracellular levels used clinically. In the present study, by considering five pharmocologically relevant biguanides as a molecular family we describe the functional effects of metformin and other biguanides on the complexes that catalyse oxidative phosphorylation in mammalian mitochondria.

## EXPERIMENTAL

### Preparation of proteins, membranes, SMPs and mitochondria

Complex I was prepared from *Bos taurus* (bovine) heart mitochondria [20], *Pichia pastoris* [21] and *Escherichia coli* [22], as described previously. SMPs (submitochondrial particles) and mitochondrial membranes were prepared from bovine heart mitochondria [20,23]. Complex IV was a by-product from the preparation of complex I; it elutes from the Q-Sepharose column at ~250 mM NaCl. Mitochondria were isolated from rat liver by the method of Chappell and Hansford [24]. F_1_F_O_-ATP synthase and the F_1_ domain were isolated from bovine mitochondria as described previously [25] using a HiLoad Superdex 200-PG column and omitting azide and 2-mercaptoethanol.

### Kinetic measurements on isolated complex I

All assays were performed at 32°C in 20 mM Tris/HCl (pH 7.2). NADH:decylubiquinone oxidoreduction was measured using 200 μM NADH and 200 μM decylubiquinone, in 0.075% soya bean asolectin (Avanti Polar Lipids) and 0.075% CHAPS (Merck Chemicals) and quantified by the absorbance of NADH (ε_340–380_=4.81 mM^−1^·cm^−1^) [20]. Catalysis was initiated by the addition of NADH, following a 2 min pre-incubation, and rates measured as the linear regression of the maximal rate (discarding any initial lag phases). Biguanides were added immediately before NADH, unless otherwise stated, and the level of inhibition did not depend on the length of pre-incubation. Initial rates for the NADH:FeCN (ferricyanide), NADH:HAR [hexaammineruthenium(III)] and NADH:paraquat reactions were measured in 100 μM NADH with 1 mM FeCN (ε_420–500_=1 mM^−1^·cm^−1^), 3.5 mM HAR or 200 μM paraquat (ε_340–380_=4.81 mM^−1^·cm^−1^) [26,27]. H_2_O_2_ formation was followed in 30 μM NADH as the catalase-sensitive horseradish peroxidase-dependent oxidation of 10 μM Amplex Red to resorufin (ε_557–620_=51.6 mM^−1^·cm^−1^), with 2 units/ml superoxide dismutase [15], or by monitoring NADH oxidation. Metformin (Cambridge Bioscience) phenformin and buformin (Santa Cruz Biotechnology) were added from aqueous stock solutions, and cycloguanil (Santa Cruz Biotechnology) and proguanil (Sigma–Aldrich) were in DMSO. Control experiments included NaCl (to maintain the ionic strength) or DMSO.

### Kinetic measurements on bovine mitochondrial membranes and SMPs

All assays were performed at 32°C in 10 mM Tris/HCl (pH 7.4) and 250 mM sucrose. NADH oxidation was measured in 100 μM NADH, and succinate oxidation in 10 mM succinate, using a coupled assay system [28]. Complex II activity was measured in 10 mM succinate and 100 μM decylubiquinone using membranes solubilized in 1% dodecylmaltoside to isolate the activity. Complex II + III activity in membranes was measured by the reduction of cytochrome *c* (ε_550–541_=18.00 mM^−1^·cm^−1^) with 120 μM oxidized horse heart cytochrome *c*, 10 mM succinate and 1 mM NaCN to inhibit complex IV. Complex IV activity was measured by the oxidation of 120 μM reduced horse heart cytochrome *c*. ATP hydrolysis was measured using a coupled assay system [23,29], using 40 μg/ml pyruvate kinase, 200 μM phosphoenol pyruvate and 50 μg/ml lactate dehydrogenase, with 4 mM MgSO_4_, 2 mM K_2_SO_4_, 4 μM rotenone and 5 μg/ml gramicidin, by monitoring the oxidation of 200 μM NADH (rotenone and gramicidin were omitted for purified enzyme measurements). To measure ATP synthesis, SMPs were incubated for 3.5 min with 200 μM ATP, 2 mM MgSO_4_, 10 mM potassium phosphate, 40 μM diadenosine pentaphosphate (AP5A, to inhibit adenylate kinase) and either 200 μM NADH or 5 mM succinate; 2 μM IF_1_ was added in some experiments but did not affect the conclusions. Reactions were quenched with 4% trifluoroacetic acid, neutralized after 20 s with 1 M Tris/SO_4_ (pH 8.1) and ATP concentrations were determined using the luciferase assay (Roche, ATP bioluminescence assay kit CLS II).

### EPR spectroscopy

EPR samples of ~11 mg/ml bovine complex I were prepared anaerobically. The samples were incubated for 15 min at 4°C with 100 mM NaCl, 100 mM metformin, 2.5 mM phenformin, or 0.35 mM proguanil, then 5 mM NADH was added and the samples frozen immediately. Spectra were recorded with 1 mW microwave power, microwave frequency 9.36–9.38 GHz, modulation frequency 100 kHz, modulation amplitude 1 mT, time constant 81.92 ms and conversion time 20.48 ms at 12 K, using a Bruker EMX X-band spectrometer with an ER 4119HS high-sensitivity cavity, maintained at low temperature by an Oxford Instruments ESR900 continuous-flow liquid helium cryostat.

### OCR and ECAR measurements on cultured human cells and isolated mitochondria

Cells [143B (cell line 8303 from the A.T.C.C.) and Hep G2 (cell line 85011430 from The Health Protection Agency)] were grown on DMEM (Dulbecco's modified Eagle's medium) supplemented with 10% FBS (Thermo Fisher Scientific) and 100 units/ml penicillin and 100 μg/ml streptomycin at 37°C in 5% CO_2_. Per well, 3×10^4^ or 1.5×10^4^ cells were plated into XF24 or XF96 (Seahorse Bioscience) plates respectively, and incubated for 12 h at 37°C in 5% CO_2_. The medium was exchanged for assay buffer containing DMEM, 4.5 g/l glucose, 1 mM pyruvate, 32 mM NaCl, 2 mM GlutaMAX, 15 mg/l Phenol Red and 20 mM Hepes (pH 7.4) and the cells placed in a CO_2_-free incubator at 37°C for 60 min. OCRs (oxygen consumption rates) and ECARs (extracellular acidification rates) were measured in a Seahorse extracellular flux analyser; 200 nM rotenone was used to inhibit complex I where stated. To calculate the normalized rotenone-sensitive OCR rates, the rotenone-insensitive rates (determined at the end of the experiment) were subtracted and the traces normalized to 100% before biguanide addition. ECAR data were normalized to 100% before biguanide addition. For the permeabilized cell experiments, cells were seeded at 3.5×10^3^ per well and incubated for 62 h at 37°C in 5% CO_2_. The medium was exchanged for assay buffer containing 3 nM plasma membrane permeabilizer ‘PMP’ (Seahorse Biosciences), 10 mM pyruvate, 10 mM malate, 220 mM mannitol, 70 mM sucrose, 10 mM KH_2_PO_4_, 5 mM MgCl, 1 mM EGTA, 0.2% fatty-acid-free BSA and 2 mM Hepes (pH 7.4) at 37°C. Cells were incubated for 15 min and a baseline was recorded for normalization, then the test compounds were added. Isolated rat liver mitochondria were plated at 20 μg per well in the same buffer but without PMP. They were adhered to the plate by centrifugation at 2000 ***g*** for 20 min at 4°C and measurements performed and normalized (after temperature equilibration) as for permeabilized cells but at 32°C.

### Analytical methods

Octanol/PBS distribution coefficients were measured by the shake-flask method [30] at 32°C and pH 7.5; they are similar to partition coefficients but refer to the protonated biguanide cations [31]. Thermo-flavin experiments were carried in an ABI 7900HT real-time PCR machine, starting at 20°C for 2 min, then stepping by 1.5°C every 30 s to 80°C [32,33]. Aliquots (20 μl) of 1 mg/ml complex I in 20 mM sodium MOPS (pH 7.5) and 200 mM NaCl (plus additives) were dispensed into a 96-well plate and sealed with optically clear seals; fluorescence intensity data were fit with a Boltzmann sigmoid using Prism. For Blue native PAGE, complex I was incubated for 24 h with or without metformin, buformin or phenformin at their complex I IC_50_ concentrations. Per lane, 21 μg was loaded on a 3–12% Novex NativePAGE pre-cast gel (Invitrogen) and run according to the manufacturer's instructions.

### Statistical methods

Experimental values are reported as means±S.E.M. for small sample sizes or as means±S.D. for large sample sizes with only technical error sources. Typically three to four replicates gave S.E.M. values of less than 5% of the mean value; numbers of replicates were increased when appropriate. Comparisons between samples are marked *P<0.05, **P<0.01 and ****P<0.0001 as determined using an unpaired two-tailed Students *t* test. Differences between measurements with *P*>0.05 were considered not significant. IC_50_ values were determined using the standard dose–effect relationship {activity (%)=100×IC_50_/(IC_50_+[inhibitor]^m^); [34] with the Hill Slope (m) set to unity for complex I} and are reported with 95% confidence intervals.

## RESULTS

### Metformin and related compounds inhibit catalysis by isolated complex I

**Figure 1:**
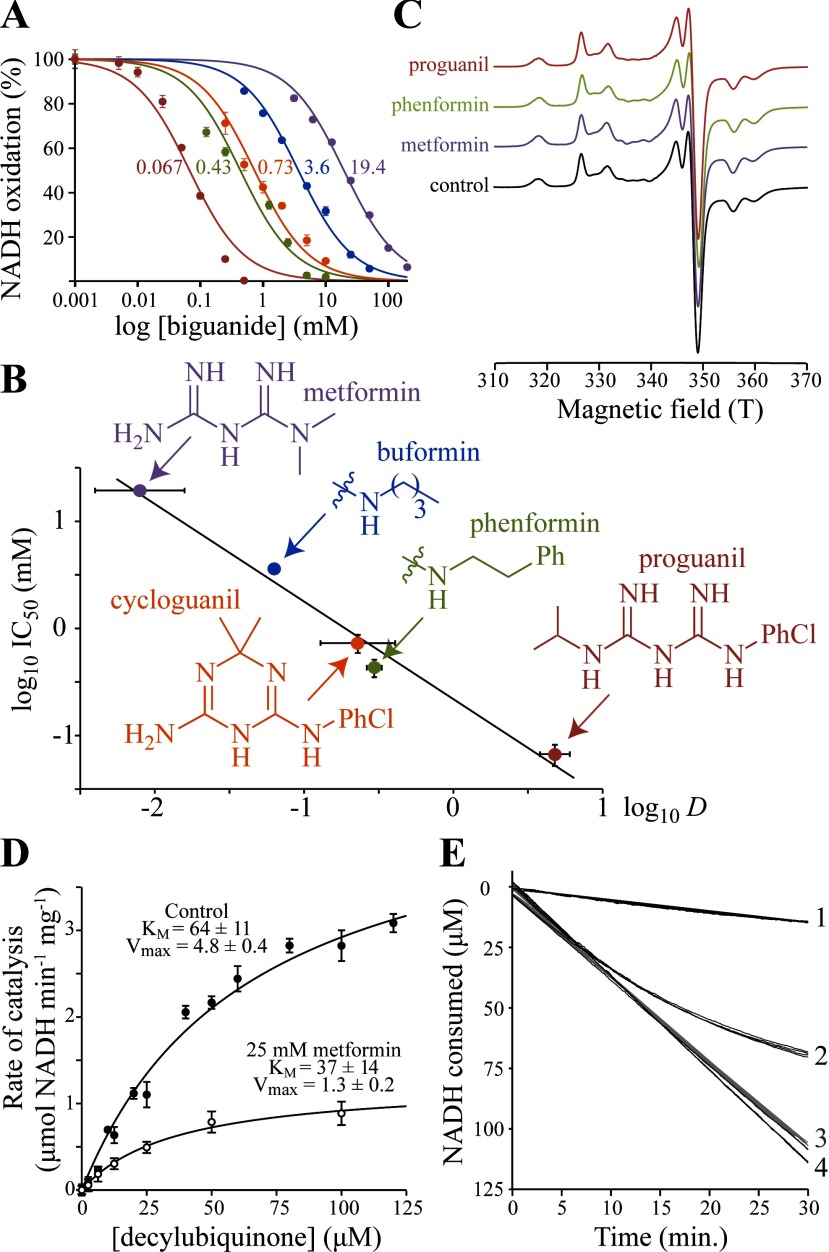
Effects of biguanides on isolated bovine complex I (**A**) Dependence of NADH oxidation on biguanide concentration, relative to a biguanide-free control. Colours are as in (**B**), and the IC_50_ values (in mM) are noted. Data points are means±S.E.M. (*n*=3–5). (**B**) Relationship between the inhibition IC_50_ values and octanol:PBS distribution coefficient (*D*) values of the biguanides. IC_50_ values are in mM with 95% confidence intervals; log *D* values are means±S.E.M. (*n*=3). Linear fit with *R*^2^=0.963. Biguanide structures shown are for the neutral forms. (**C**) 12 K EPR spectra of the FeS clusters in complex I in the presence and absence of biguanides. Ph, phenyl; PhCl, *para*-chlorophenyl. (**D**) The effect of 25 mM metformin on NADH:decylubiquinone oxidoreduction, presented as the measurement of *K*_M_ for decylubiquinone. The data (means±S.E.M.; *n*=3) were fit using the Michaelis–Menten equation. (**E**) Metformin inhibition of NADH oxidation by SMPs (4.5 μg protein/ml). Trace 1, 100 mM metformin added before initiation of catalysis by 200 μM NADH at t=0. Trace 2, catalysis initiated by 200 μM NADH 10 min before the addition of 100 mM metformin at t=0. Traces 3 and 4, controls for 1 and 2, with NaCl instead of metformin. Four traces are overlaid for each condition.

We used complex I isolated from *B. taurus* (bovine) heart mitochondria [20] to test the ability of metformin to inhibit NADH:decylubiquinone oxidoreduction (Figure 1A); decylubiquinone is a less-hydrophobic analogue of the physiological substrate, ubiquinone-10. The metformin IC_50_ value of 19.4±1.4 mM shows that metformin is only a weak inhibitor of complex I catalysis, in general agreement with previous data from immunoprecipitated complex I (IC_50_=66 mM) [8]. Inhibition of the complexes I from the yeast *P. pastoris* and the bacterium *E. coli* was also observed, with IC_50_ values of 22.6±4.3 mM and 60.7±8.5 mM respectively, indicating that the biguanide-binding site is formed by the ‘core’ subunits that are common to all complexes I, not by mammalian-specific subunits [14]. Importantly, pre-incubating complex I in high concentrations of metformin before measuring its activity in lower concentrations showed that metformin binding is reversible, and 24 h incubations followed by Blue native PAGE analyses revealed no loss of global structural integrity. Subsequently, we considered the two additional anti-diabetic biguanides, phenformin and buformin, and the two anti-malarial biguanides, cycloguanil and proguanil. Figure 1(A) shows that all five biguanides inhibit complex I catalysis, with the more hydrophobic biguanides inhibiting more strongly, suggesting they bind in an amphipathic region of the enzyme. Then, measuring the hydrophobicity of each biguanide (as *D*, the octanol:PBS distribution coefficient at pH 7.5 [30]) revealed a striking linear relationship between the log *D* and log IC_50_ values (Figure 1B). Finally, the guanidinium ion is a close relative of metformin, and we found that it inhibits (IC_50_=25.8±3.9 mM) very similarly to metformin, in contrast with the results of a previous report that guanidinium has no inhibitory effect [35].

### Biguanides inhibit ubiquinone reduction, but not competitively

**Figure 2:**
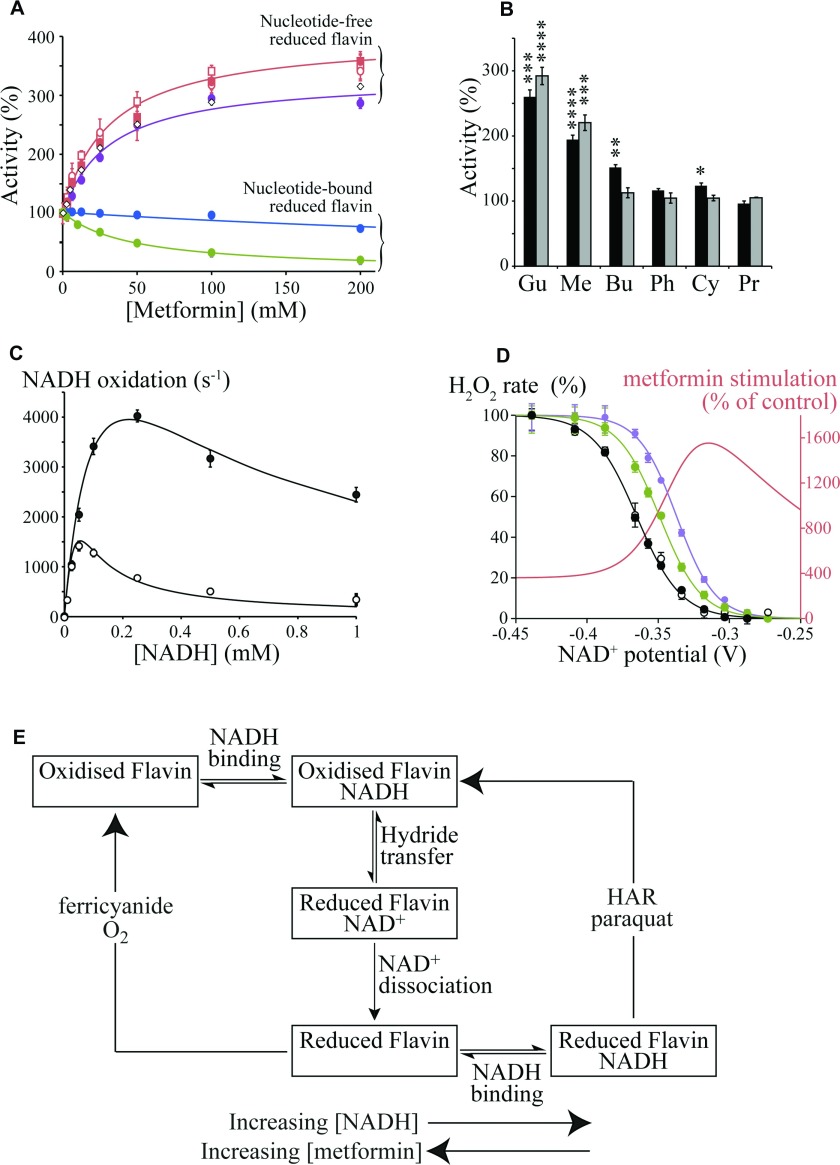
Biguanide effects on the flavin site of complex I (**A**) Effects of metformin on flavin site reactions that require nucleotide binding to the reduced flavin [NADH:HAR (blue) and NADH:paraquat (green) oxidoreduction] [27], and nucleotide-free reduced flavin [NADH:FeCN (purple) and NADH:O_2_ (red) oxidoreduction] [26]. NADH:O_2_ oxidoreduction (H_2_O_2_ production) was detected directly (by NADH oxidation, circles) or as H_2_O_2_ by the Amplex Red assay (squares) [15], with/without (open/closed symbols respectively) 1 μM rotenone. Data for H_2_O_2_ production by subcomplex Iλ are shown as open diamonds (◇). Data points are means±S.E.M., *n*=3–5. (**B**) Stimulation of flavin-site reactions, measured at the inhibitory IC_50_ concentrations (black, H_2_O_2_ production measured using Amplex Red; grey, NADH:FeCN oxidoreduction). Gu, 25 mM guanidinium; Me, 25 mM metformin; Bu, 5 mM buformin; Ph, 0.5 mM phenformin; Cy, 0.7 mM cycloguanil; Pr, 0.05 mM proguanil. Data are presented as means±S.E.M., *n*=3–5. (**C**) NADH-dependence of NADH:FeCN catalysis by isolated bovine complex I. ●, 100 mM metformin; ○, control. Data points are means±S.E.M., *n*=3–5 and data were fit as described previously [26] with *K*_M_^NADH^=220 μM, *k*_cat_^NADH^=1500 s^−1^, *k*_cat_^FeCN^=3.7×10^7^ M^−1^·s^−1^ and *K*^NADH^_Red_ (the dissociation constant for NADH binding to the reduced flavin)=*K*^NADH^_Semi_=11 μM (control) or 181 μM (metformin). (**D**) Dependence of H_2_O_2_ production on the NAD^+^ potential, set by the Nernst equation with 30 μM NADH and variable NAD^+^ [15]. Control [with (●) and without (○) 200 mM NaCl], 20 mM phenformin (green) and 200 mM metformin (purple). Data points are means±S.E.M., *n*=3–5. The stimulation of H_2_O_2_ production by metformin (red) is also represented as the non-normalized percentage activity relative to the control (see **A** for the effect of metformin in NADH only). (**E**) Simplified scheme illustrating how metformin affects the different flavin site reactions differently. The boxes represent different states of the complex I flavin site, with the flavin oxidized or reduced, and with or without NADH or NAD^+^ bound.

To narrow down the location at which biguanides bind to complex I, we tested their effects on different steps in the catalytic cycle: NADH oxidation by the flavin mononucleotide, intramolecular electron transfer along the chain of FeS clusters, and ubiquinone reduction [14]. First, metformin was found to stimulate (see below and Figure 2), not inhibit, the NADH:FeCN oxidoreduction reaction. The NADH:FeCN reaction is widely used to test the rate of NADH oxidation by complex I, because the whole reaction is localized exclusively at the flavin site [26]. Furthermore, even extremely high concentrations of metformin only affected the thermal stability of the flavin site slightly, showing that the flavin environment is not significantly altered (the thermal stability is measured as a melting temperature that varies from 56.3±0.4 in 200 mM NaCl to 55.3±0.1 in 200 mM metformin). We conclude that the biguanides do not inhibit complex I by inhibiting NADH oxidation. Secondly, Figure 1(C) shows that biguanides do not affect the ‘fingerprint’ EPR spectra of the FeS clusters of NADH-reduced complex I [36], suggesting they do not inhibit intramolecular electron transfer. Thirdly, Michaelis–Menten analyses [37] were used to determine whether metformin is a competitive inhibitor towards ubiquinone substrates (Figure 1D). Because both the *K*_M_ and *k*_cat_ values for decylubiquinone are altered by metformin we conclude that biguanides do not bind competitively in the ubiquinone-binding site. Instead, metformin is a reversible non-competitive inhibitor, that binds to complex I whether ubiquinone is bound or not. We conclude that metformin inhibits a rate-determining step that is mechanistically coupled to ubiquinone reduction. Consequently, the biguanides constitute a hitherto unknown class of complex I inhibitor, consistent with them being positively charged and relatively hydrophilic molecules that do not resemble the highly hydrophobic uncharged canonical complex I inhibitors, such as rotenone and piericidin A.

### Metformin interacts with ‘deactive-like’ conformations of complex I

SMPs are robust, inverted and tightly coupled vesicles of the inner membrane of bovine heart mitochondria [23]. NADH oxidation by complex I in SMPs does not require a hydrophilic ubiquinone because ubiquinone-10 is present, and ubiquinol-10 is reoxidized by the respiratory chain. Therefore SMPs maintain steady-state NADH oxidation much longer than isolated complex I. Figure 1(E) shows that NADH oxidation by SMPs is inhibited immediately if metformin is added before catalysis is initiated (trace 1 compared with trace 3). However, if metformin is added during catalysis then inhibition develops slowly (trace 2), taking more than 30 min to attain the same level. Therefore inhibition depends strongly on the catalytic status of complex I. The first matrix loop of subunit ND3 in complex I, containing Cys^39^, is a coupling element in the amphipathic region between the redox and proton-transfer domains; it reaches up to the hydrophilic domain from the membrane domain, pinning them together [14,38]. In resting mammalian complex I the loop gradually adopts an altered ‘open’ conformation (often referred to as the deactive state) in which Cys^39^ can be derivatized [39]. Open-loop conformations are catalytically inactive, and the redox and proton-transfer domains are no longer coupled [40]; when substrates become available the loop ‘closes’ and the enzyme returns to catalysis. Figure 1(E) suggests that metformin traps the enzyme in a deactive-like open-loop conformation, by binding in the amphipathic region at the interface of the hydrophilic and membrane domains, where redox-driven processes initiate proton translocation. Thus biguanides may inhibit complex I by stabilizing an inactive conformation, similar to the way in which guanidinium inhibits voltage-gated potassium channels by stabilizing a closed channel conformation [41].

### Biguanide effects on the flavin site and H_2_O_2_ production

Complex I catalyses electron transfer from NADH to hydrophilic electron acceptors by two distinct mechanisms, both localized uniquely at the flavin site. The NADH:FeCN and NADH:O_2_ reactions proceed via two sequential steps in which NADH reduces the flavin and dissociates, and then the electron acceptor reacts directly (see Figure 2E, left-hand reaction) [26]; for the NADH:HAR and NADH:paraquat reactions NADH reduces the flavin, but the electron acceptor reacts only if a nucleotide is bound in the reduced flavin site (see Figure 2E, right-hand reaction) [27]. Note that although NADH:O_2_ oxidoreduction produces superoxide, we detect the H_2_O_2_ formed following superoxide dismutation, and thus refer to ‘H_2_O_2_ production’. Data showing the effects of biguanides on these different reactions are shown in Figure 2. Figure 2(A) shows that metformin stimulates the rates of the two reactions (the NADH:FeCN and NADH:O_2_ reactions) that rely on oxidation of the nucleotide-free reduced flavin [15,26], but, conversely, it inhibits the rates of two reactions [the NADH:HAR and NADH:paraquat reactions] that rely on oxidation of the nucleotide-bound reduced flavin [27]. Dose–response stimulatory effects were also observed on the NADH:FeCN reactions catalysed by the complexes I from *P. pastoris* and *E. coli* (the rates increased to 166±1% and 144±2% respectively, at 200 mM metformin) and the dose–response effects of guanidinium and metformin on H_2_O_2_ production were very similar (the rate maxima were 386±34% and 338±41% with EC_50_ values of 9.3 to 21.9 mM and 21.3 to 56.9 mM respectively). Stimulation of H_2_O_2_ production by complex I by guanidinium has also been described previously [35,42]. Importantly, several observations clearly demonstrate that the effects of biguanides on the flavin site and on ubiquinone reduction are discrete. The stimulatory effect of metformin on H_2_O_2_ production was not affected by rotenone, a canonical Q-site inhibitor, and the same effects were observed in the isolated hydrophilic domain subcomplex (Iλ) of bovine complex I [43], that lacks a functional ubiquinone-binding site (see Figure 2A). Furthermore, Figure 2(B) shows that the most hydrophobic biguanides (that inhibit ubiquinone reduction most) affect the flavin least, revealing clearly opposing trends in efficacy between the two effects.

Two conditions that control the rate of H_2_O_2_ production at the flavin site are the nucleotide concentration (since NADH binding to the reduced flavin blocks O_2_ access [26]), and the redox status of the flavin, set by the ratio of NAD^+^ to NADH [15]. Figure 2(C) shows the NADH-dependence of the NADH:FeCN reaction in the presence and absence of 100 mM metformin; metformin alleviates the inhibition of the reaction at high NADH concentrations by decreasing nucleotide occupancy of the reduced flavin site (see Figures 2C and 2E). Similarly, the decreased nucleotide occupancy of the reduced flavin site explains how metformin slows the rates of NADH:HAR and NADH:paraquat oxidoreduction (see Figures 2A and 2E). Indeed, we estimate (by using a data-fitting method described previously [26], see fitted lines in Figure 2C), that the dissociation constant for NADH to the reduced flavin is increased by ~20 times in 100 mM metformin. Figure 2(D) shows the effects of metformin and phenformin on the NAD^+^ potential-dependence of isolated complex I (describing the flavin midpoint potential in accordance with the Nernst equation), normalized to the rate in NADH only. The potential dependence shifts in the presence of biguanide so that H_2_O_2_ production is especially increased at higher potentials (when the NAD^+^ pool is more oxidized). Related behaviour has been observed previously for guanidinium [35,44]. The flavin midpoint potential was found to shift by +15–25 mV, suggesting that biguanides stabilize the reduced flavin, relative to the oxidized state. *In vivo*, the direct effects of metformin are compounded by increased reduction in the NADH pool due to inhibition of complex I catalysis, providing the possibility of more than 20-fold increases in H_2_O_2_ production from the complex I flavin site.

Currently, there are few direct data by which to assess the effects of metformin on mitochondrial or cellular H_2_O_2_ production. We used the fluorescence of resorufin to follow H_2_O_2_ production by isolated rat skeletal muscle mitochondria respiring on glutamate and pyruvate (to produce NADH) in the presence of ATP and rotenone (to build Δ*P*), and found the rate to be stimulated by 270±34% and 360±35% in 50 and 100 mM metformin respectively. However, these extramitochondrial concentrations are very high and we are unable to control the intramitochondrial concentration or conditions, so the results are not quantitatively meaningful. Previously, incubation of isolated liver mitochondria with metformin revealed a small, but progressive, increase in H_2_O_2_ production [45], and biguanides were observed to increase ROS production in cells [8]; it has further been suggested that metformin-stimulated reactive oxygen production provides an alternative route to the activation of AMPK [46,47]. In contrast, metformin has been reported to decrease paraquat-induced H_2_O_2_ production in cells [48]; paraquat is reduced by complex I, and then reacts with O_2_, accelerating H_2_O_2_ production by redox cycling [27]. Figure 2(E) explains how metformin brings about these opposing effects: metformin induced decreased NADH binding to the reduced flavin site which increases ‘native’ H_2_O_2_ production, but decreases the paraquat-stimulated rate.

### Biguanide interactions with respiratory complexes II, III and IV

**Figure 3:**
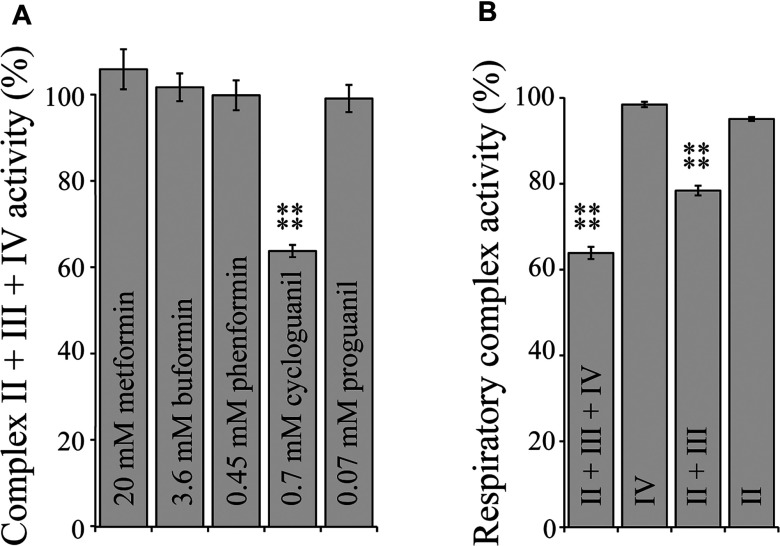
Biguanide interactions with respiratory complexes II, III and IV (**A**) Rates of complex II + III + IV activity in bovine SMPs measured by a coupled enzyme assay [28] in the presence of biguanide concentrations equivalent to IC_50_ for NADH:decylubiquinone catalysis. Results are means±S.E.M. as a percentage of the biguanide-free control, *n*=3–4. (**B**) Rates of respiratory complex activity in the presence of 0.7 mM cycloguanil as a percentage of their respective cycloguanil-free controls. ****P<0.0001.

Complex I has been proposed as the major respiratory-chain target of metformin [7,49], but inhibition of complexes II and IV have also been reported in rat liver mitochondria [50]. We tested the inhibitory effects of all five biguanides, at concentrations equivalent to their IC_50_ values for isolated complex I, on the succinate:O_2_ activity of complexes II + III + IV in SMPs and found that only cycloguanil had a significant effect (see Figure 3A). Inhibition of complex III was found by a process of elimination; succinate:cytochrome *c* oxidoreduction by NaCN-inhibited SMPs was inhibited, yet neither succinate:decylubiquinone oxidoreduction by solubilized membranes, or cytochrome *c* oxidation by isolated cytochrome *c* oxidase were affected (Figure 3B). The IC_50_ of cycloguanil on complex III was subsequently determined to be 2.48±0.21 mM, more than three times higher than the equivalent value for complex I.

### Effects of biguanides on F_1_F_0_-ATPase

**Figure 4:**
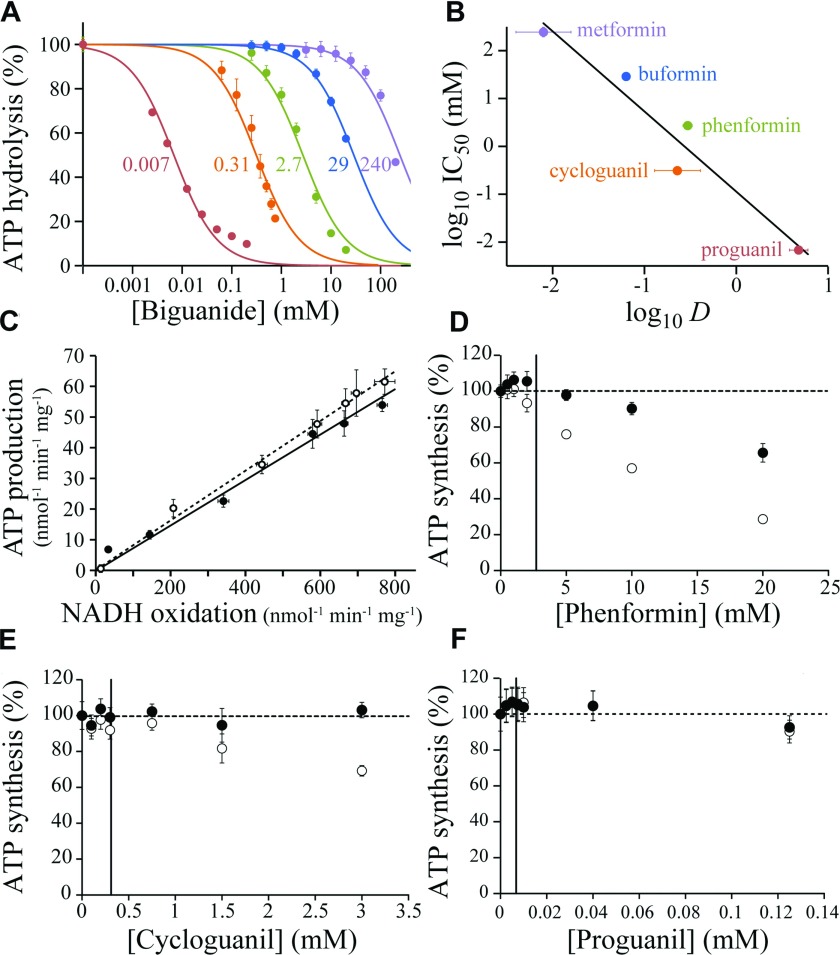
Biguanide inhibition of F_1_F_0_-ATP synthase in bovine SMPs (**A**) Inhibition of ATP hydrolysis by SMPs (colours as in **B**). Data points are means±S.E.M., *n*=3–5, and IC_50_ values are indicated in mM. (**B**) Relationship between the IC_50_ values for ATP hydrolysis and the log *D* values. IC_50_ values are in mM with 95% confidence intervals; log *D* values are means±S.E.M., *n*=3. Linear fit with *R*^2^=0.948. (**C**) Relative inhibition of ATP production in SMPs in the presence of piericidin A (○) or metformin (●). ATP synthesis was driven using 200 μM NADH; NADH oxidation was measured spectroscopically, and the concentration of ATP determined after 3.5 min (during this time a linear rate is observed). NADH oxidation rates were adjusted using 0–50 nM piericidin A or 0–250 mM metformin, with the ionic strength kept constant at 250 mM using NaCl. (**D**–**F**) Dose-dependent effects of biguanides on succinate-driven ATP production. Data points are means±S.E.M., *n*=3, as a percentage of ATP production in the absence of biguanide. ATP concentrations were determined after 3.5 min (○) then corrected for the rate of succinate oxidation, detected spectroscopically using a coupled assay system [28] (●). The IC_50_ values for hydrolysis are marked with vertical lines.

The effects of biguanides on ATP hydrolysis by F_1_F_O_-ATPase were tested in SMPs by using a coupled assay to detect the formation of ADP [23,29]. Figure 4(A) shows that all five biguanides inhibit ATP hydrolysis, and intriguingly, a similar relationship between efficacy and hydrophobicity is observed for ATPase as for complex I (compare Figures 1B and 4B). To attempt to determine the location of the biguanide-binding site on ATPase we compared inhibition of ATP hydrolysis in SMPs to inhibition of hydrolysis by the isolated F_1_ domain. At the SMP IC_50_ values (marked on Figure 4A) buformin and phenformin inhibited the isolated F_1_ domain by 74±3% and 32±7.5% respectively, but no significant inhibition could be observed for cycloguanil or proguanil. However, inhibition of ATP hydrolysis in SMPs by proguanil was confirmed to be a primary effect on ATPase by testing the isolated intact enzyme; indeed, the observed IC_50_ value of 0.67±0.1 μM for isolated ATPase is lower than for SMPs (6.8±0.7 μM). This complicated picture may be rationalized by either a single binding site in the interfacial region that is altered upon extraction of the enzyme from the membrane and only partially retained in the isolated F_1_ domain, or by the presence of more than one binding site, as in complex I.

The effects of biguanides on ATP synthesis were measured by following ATP production by SMPs respiring on either NADH or succinate. First, the inhibition of NADH-driven ATP synthesis by metformin was compared with its inhibition by piericidin A, a highly specific complex I inhibitor (Figure 4C). In this assay, the effects of metformin (up to 250 mM) and piericidin A are essentially identical, indicating that inhibition of ATP synthesis results from inhibition of complex I, with little effect on either ATP synthase itself, or on proton leak or uncoupling of the proton motive force. Subsequently, similar experiments using succinate oxidation to drive ATP synthesis (since it is much less affected by biguanides) were used to focus on the ATP synthase reaction (Figures 4D–4F). Succinate oxidation was monitored, and used to correct the amount of ATP produced for the inhibition of respiration that is observed. No inhibition of ATP synthesis by 15 mM buformin or 100 mM metformin was observed; higher concentrations of metformin could not be tested because high ionic strength interferes with our succinate oxidation assay [28]. For phenformin, the concentration range could be extended substantially above the IC_50_ for ATP hydrolysis (Figure 4D); inhibition of synthesis is observed at higher concentrations, although the IC_50_ is an order of magnitude higher than for hydrolysis. Figures 4(E) and 4(F) show that neither cycloguanil or proguanil affect ATP synthesis at concentrations considerably higher than their IC_50_ values for ATP hydrolysis, so they are unidirectional inhibitors of ATPase. This observation is consistent with the specificity of cycloguanil and proguanil for just one of two putative binding sites.

### Selective cellular and mitochondrial uptake of biguanides

**Figure 5:**
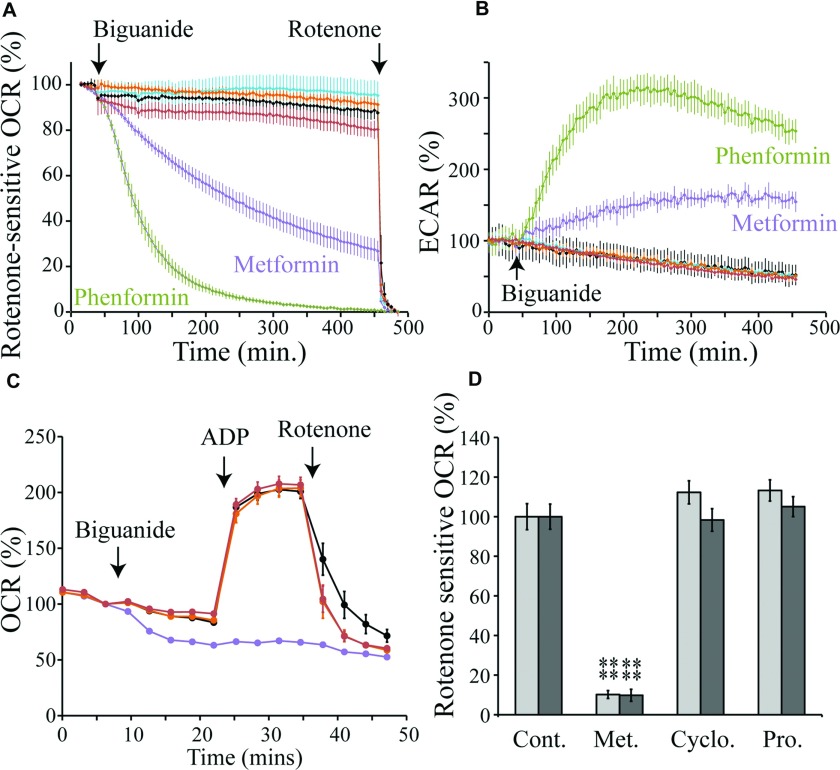
Selective uptake of biguanides into mitochondria (**A**) Effects of biguanides on the rotenone-sensitive OCR of Hep G2 cells. The traces are the means±S.D. of multiple traces. The biguanide concentrations are equal to the complex I IC_50_/10 values. Purple, 1.9 mM metformin; green, 0.04 mM phenformin; orange, 0.07 mM cycloguanil added in DMSO; red, 0.007 mM proguanil in DMSO; black, control (no biguanide); cyan, control (DMSO). (**B**) The effects of biguanides on the ECAR of Hep G2 cells. Conditions and colours as in (**A**). (**C**) Mitochondrial respiratory coupling test on 143B cells permeabilized with 2 nM PMP, respiring on pyruvate and malate and using biguanides at concentrations equal to their complex I IC_50_/10 values. Black, control; purple, 2 mM metformin; orange, 0.07 mM cycloguanil; red, 0.007 mM proguanil. Data points are means±S.E.M., *n*=5–6. (**D**) Rates of rotenone-sensitive ADP-stimulated NADH-linked respiration in permeabilized 143B cells (light grey) and rat liver mitochondria (dark grey) after 15 min of incubation with 2 mM metformin (Met.), 0.07 mM cycloguanil (Cyclo.) or 0.007 mM proguanil (Pro.). Values are mean percentage of the control±S.E.M., *n*=5–6. ****P< 0.01. Cont. control.

The two antimalarial biguanides, proguanil and cycloguanil, are good inhibitors of complex I, but we could find no clinical reports for either being associated with increased risk of lactic acidosis. In contrast, the anti-diabetic drugs metformin, phenformin and buformin are weaker inhibitors and they are associated with lactic acidosis [17]. This comparison suggests that proguanil and cycloguanil do not access complex I in cells, so we tested the uptake of biguanides into the mitochondria of two human cells lines (143B osteosarcoma and Hep G2 hepatocarcinoma cells) by monitoring inhibition of complex I. To monitor complex I catalysis we measured OCRs (rotenone-sensitive OCRs are proportional to the rate of complex I catalysis) and ECARs (representing the export of lactate from the cells, which increases due to increased glycolysis as respiration is inhibited) in a Seahorse XF analyser. The two cell lines behaved similarly, and Figures 5(A) and 5(B) show their response to biguanide concentrations equal to one tenth of their isolated complex I IC_50_ values. Uptake of phenformin is relatively rapid, reaching completion after approximately 6 h, whereas metformin accumulates more slowly, and its inhibition is still developing at the end of the experiment. In contrast with the considerable effects of metformin and phenformin, neither proguanil nor cycloguanil exhibited any substantial effect on either the rotenone-sensitive OCR or the ECAR after 6 h of treatment, so we consider that they do not access complex I. To understand whether they are blocked by the plasma or mitochondrial membrane, plasma-membrane-permeabilized 143B cells and isolated rat liver mitochondria, respiring on pyruvate and malate, were analysed. Figure 5(C) shows data for permeabilized 143B cells. The normalized rotenone-sensitive OCRs from Figure 5(C), and data from a parallel experiment using isolated rat liver mitochondria, are compared in Figure 5(D). Our data clearly distinguish the anti-diabetic biguanides metformin and phenformin, which inhibit complex I in cells by entering mitochondria, from the anti-malarial biguanides cycloguanil and proguanil, which do not. Because cycloguanil and proguanil are the most hydrophobic biguanides tested (see Figure 1B), this result indicates that biguanide uptake into mitochondria is protein-mediated, and does not occur by passive diffusion across the membrane.

### Biguanides accumulate reversibly in mitochondria according to the membrane potential

**Figure 6:**
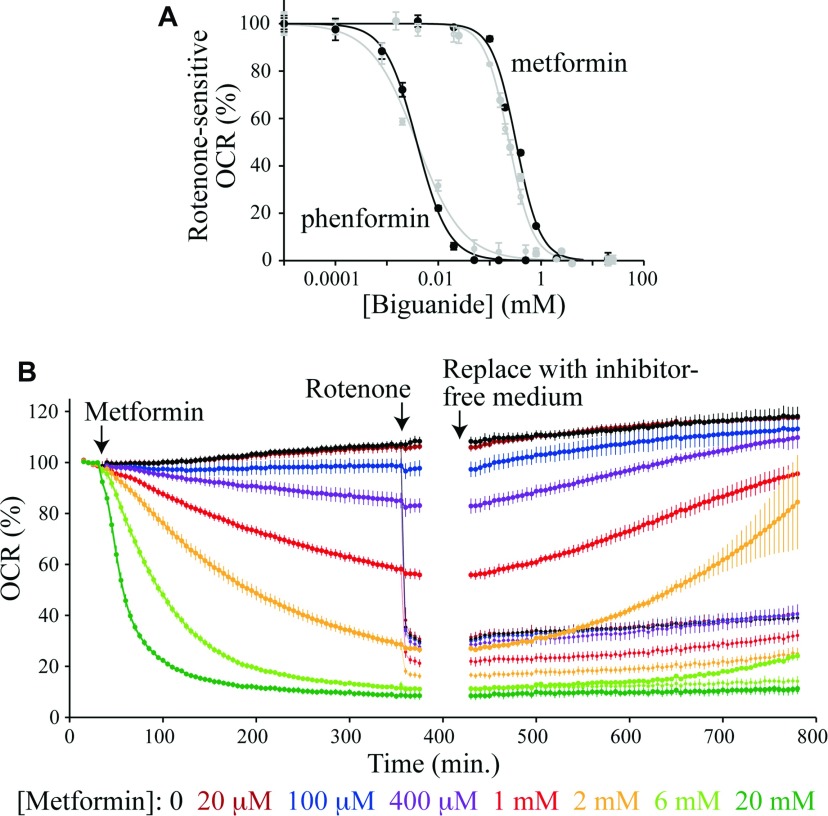
Biguanide effects on cells in culture: accumulation and reversibility (**A**) The normalized rotenone-sensitive OCR 6 h after the addition of metformin or phenformin to Hep G2 (black) or 143B (grey) cells. Results are means±S.E.M., *n*=3–4. The IC_50_ values (with 95% confidence intervals) are metformin, 240±10 μM (143B) and 330±20 μM (Hep G2); phenformin, 3.9±1.0 μM (143B) and 3.8±0.4 μM (Hep G2). (**B**) Metformin inhibition of the OCR by Hep G2 cells. Results are means of multiple traces±S.E.M., *n*=3–4. Rotenone (200 nM) was added to half of the samples part way through the experiment; pairs of traces with and without rotenone are coloured the same. Then, the assay medium in each experiment was exchanged for a metformin and rotenone-free medium. All data were measured using a Seahorse Extracellular Flux Analyzer at 37°C.

Biguanides are positively charged so they are expected to accumulate in the mitochondria of cells in response to the potential difference across the inner membrane (the potential difference is the ‘charge’ component of the protonmotive force). Each 60 mV of potential difference drives the concentration of the positive ion to 10-times higher inside the mitochondrion than outside. For a typical potential difference of 120–150 mV the concentration inside the mitochondrion could therefore be 100 to 300-times higher than outside. Figure 6(A) presents the effective IC_50_ values for metformin and phenformin on 143B and Hep G2 cells after 6 h of exposure to the biguanide. The OCRs were normalized to their values before biguanide addition, and monitored throughout the experiment; rotenone-insensitive rates were subtracted, and all rates after 6 h compared with a biguanide-free control. Figure 6(B) shows that accumulation of the biguanides (especially metformin) into the cells/mitochondria is slow, requiring many hours, so our cellular IC_50_ values are underestimates, and they are also confounded by a negative-feedback loop (complex I catalysis forms the membrane potential that accumulates the biguanides into mitochondria and the biguanides inhibit complex I catalysis). The cellular IC_50_ values are 237±13 μM and 325±25 μM for metformin, for 143B and Hep G2 cells respectively, and 3.81±1.12 μM and 3.80±0.38 μM for phenformin. By comparison with the isolated complex I IC_50_ values, these values equate to 60 to 80-fold accumulation of metformin, and 120-fold accumulation of phenformin. Our IC_50_ values are comparable with those reported for primary hepatocytes after 24 h of incubation, and considerably lower than previous values reported for Hep G2 cells [8]. In order to observe the degree of reversibility of accumulation, cells were incubated for 6 h with metformin, then the medium was exchanged for fresh metformin-free medium, and the recovery of the OCR was monitored (Figure 6B). Metformin inhibition, by all but the highest concentrations (which presumably induce cell death), was gradually alleviated, mirroring the onset of inhibition and demonstrating its reversible nature. In contrast, rotenone inhibition was not reversible, and the fact that excessive metformin inhibition decreases oxygen consumption below the rotenone-sensitive level suggests either increased cell death and/or a secondary effect. Notably, both the onset and offset of metformin inhibition are much slower in cells than in isolated complex I, suggesting that they are limited by slow transport processes.

## DISCUSSION

### The non-specificity of biguanide interactions

We have found that biguanides interact with two separate sites on complex I; one site perturbs the reactivity of the flavin, the other inhibits catalysis. We have also found that biguanides inhibit ATP hydrolysis by ATP synthase, and that some biguanides inhibit only hydrolysis, not synthesis. The other three mammalian oxidative phosphorylation complexes are not significantly affected by biguanides except for a comparatively weak inhibition of complex III by cycloguanil. Dipeptidylpeptidase 4 [11], AMP deaminase [12] and hexokinase II [13] are also inhibited by metformin. Dihydrofolate reductase is inhibited by cycloguanil [19], proguanil affects the mitochondrial membrane potential in *Plasmodium* [18], and metformin disrupts folate-related one-carbon metabolic pathways [51,52]. Recently, mitochondrial glycerophosphate dehydrogenase has also been reported to be inhibited by metformin, and this effect has been proposed as the origin of metformin's ability to suppress gluconeogenesis [3]. Importantly, this report has revealed the possibility of a dual effect for metformin on NADH oxidation: inhibiting the extramitochondrial oxidation of NADH produced by glycolysis, as well as the oxidation of NADH produced in (or shuttled into) the mitochondrial matrix.

Clearly, biguanides interact with many different enzymes and some insight into this non-specificity is provided by the structural relationship between biguanides and guanidinium. At high concentrations guanidinium is a protein denaturant, but at low concentrations its trigonal-planar structure promotes a diverse set of protein interactions: it forms ion pairs with negatively charged residues and cation-π interactions with aromatic residues, is a hydrogen-bond donor, can mimic and displace the arginine side chain, and also disrupt hydrophobic interfaces [53,54]. In the PDB ~50% of structurally defined guanidiniums interact with carboxylate side chains, and ~60% with peptide carbonyls, so carboxylate residues are good candidates for future experiments to identify specific interacting residues. Within the complexes of oxidative phosphorylation, complex I and ATP synthase may be particularly susceptible to biguanide inhibition because of catalytic conformational mobility in their domain interfaces.

### Unusual features of biguanides as complex I and ATPase inhibitors

Piericidin is a canonical inhibitor of complex I; it competes for the ubiquinone-binding site [38] and is an uncharged, aromatic and highly hydrophobic molecule. In contrast, we have shown that biguanides do not compete with ubiquinone, and they are positively charged and relatively hydrophilic molecules. Metformin, in particular, inhibits far more weakly than piericidin A and other canonical inhibitors, and, crucially, its inhibition is reversible. In a pharmacological context, this ‘fast and weak’ inhibition that responds rapidly to the cellular conditions (allowing catalysis to be re-established immediately once the conditions become favourable) and the positive biguanide charge, which establishes the negative-feedback loop between catalysis and accumulation that is likely critical in balancing desired effects with avoidance of lactic acidosis, combine to lend metformin unique therapeutic properties as a complex I inhibitor. Furthermore, metformin inhibits by trapping complex I in a deactive-like conformation, most likely by binding in the amphipathic region at the interface of the hydrophilic and membrane domains, close to the matrix loop of subunit ND3. The rapid binding of metformin to the deactive state may be relevant to therapeutic strategies for the inhibition of complex I in ischaemic tissue (see below). Finally, metformin amplifies the effects of complex I inhibition on stimulating H_2_O_2_ production, as an alternative route to the activation of AMPK [46,47].

The site(s) at which biguanides bind to ATP synthase, and their inhibitory mechanism(s), are currently unknown, but the two antimalarial biguanides, cycloguanil and proguanil, stand out from the multitude of known ATP synthase inhibitors by their unidirectional behaviour, being inhibitors of hydrolysis but not synthesis. Except for the mitochondrial inhibitor protein of ATP hydrolysis, IF_1_ [55], we were only able to find one extant report of any other such inhibitor [56], with the compounds described being a series of substituted guanidine derivatives.

### Biguanide uptake into cells and mitochondria

Comparison of typical blood serum levels for the biguanides with their complex I IC_50_ values initially presented a conundrum. Metformin is found in sera at ~2000-times lower concentration than its complex I IC_50_ value (~IC_50_/2000), whereas phenformin (the biguanide with the highest risk of lactic acidosis) is in sera at ~IC_50_/1000, and proguanil and cycloguanil (with no reported lactic acidosis risk) are present at ~IC_50_/10 and ~IC_50_/5000 respectively [57]. We have explained these relationships by showing that proguanil and cycloguanil do not inhibit complex I in human cells because they are excluded from mitochondria; it is known that they are taken up into the cytoplasm of hepatocytes, since proguanil is converted into cycloguanil by intracellular P450s [58,59]. The organic cation transporter OCT1 (organic cation transporter 1), expressed to high levels in liver and kidney, is considered the main plasma-membrane metformin transporter [5,60], consistent with tissue-specific accumulation of biguanides in the liver and the lack of other systemic effects. However, ablating OCT1 in cultured ovarian cancer cells prevented activation of AMPK only by metformin, not by phenformin [61], suggesting that different biguanides may rely on different transporters. We have shown that proguanil and cycloguanil are unable to traverse biological membranes unaided, and it is very likely that a similar conclusion applies to phenformin, which is of similar hydrophobicity to cycloguanil (see Figure 1). It is possible that the human OCTN1 transporter, which has been located in mitochondria [62], is responsible for the mitochondrial uptake of anti-diabetic biguanides [63].

Although it is not clear how much complex I inhibition is required to lower gluconeogenesis clinically, it is clear that serum levels of metformin and phenformin are much too low, themselves, to have any effect. Thus it is important to realize that accumulation of positively charged molecules in the mitochondrial matrix, in response to the protonmotive force across the inner mitochondrial membrane, is a well-described physical phenomenon that raises the intramitochondrial concentration far above the external concentration; this effect has been discussed previously for biguanides [7,8]. In the present study, we inferred intramitochondrial biguanide concentrations from levels of complex I inhibition, and observed approximately 100-fold accumulation in the mitochondria of cultured cells, relative to in the external medium. *In vivo*, combined plasma and mitochondrial membrane potentials of 180 mV will (eventually, subject to transport processes) accumulate metformin and phenformin by 1000-fold in the mitochondrial matrix, relative to in serum. This represents an increase from the serum concentrations of ~10 μM and ~200 nM respectively [57] to mitochondrial concentrations of 10 mM and 200 μM, sufficient to inhibit catalysis by ~25%. Furthermore, metformin concentrations in the gut and hepatic portal vein following oral administration are higher than in the rest of the body [5]. Our results are consistent with (but do not prove) complex I inhibition in hepatocytes as the origin of the anti-hyperglycaemic activity of metformin, and with the concept that, *in vivo*, inhibition of oxidative phosphorylation by biguanides is heterogeneous across different tissues, varying not only with tissue drug concentrations, but also with factors that influence transport across cytoplasmic and mitochondrial membranes.

### ATP hydrolysis in *Plasmodium*

As anti-malarials, cycloguanil inhibits *Plasmodium* dihydrofolate reductase [19], and proguanil acts synergistically with atovaquone, an inhibitor of *Plasmodium* respiratory complex III [18]. *Plasmodium* (spp.) rely on their respiratory chain (which does not contain complex I) to support pyrimidine biosynthesis, and to support the essential mitochondrial membrane potential; proguanil inhibits an alternative (respiratory-chain independent) pathway for generating the membrane potential that has been proposed to involve ATP hydrolysis by the *Plasmodium* ATPase [18]. Although *Plasmodium* (spp.) appear to lack some ATPase subunits, and we have not tested proguanil on *Plasmodium* ATPase itself, our results suggest that proguanil may inhibit ATP hydrolysis in *Plasmodium* directly, thereby, in combination with atovaquone, knock out the essential *Plasmodium* membrane potential. Notably, mammalian cells that lack mitochondrial DNA sustain a membrane potential through electrogenic exchange of ATP and ADP, coupled to ATP hydrolysis by an incomplete version of ATP synthase [64], demonstrating that direct proton-translocation by ATPase may not be essential in this role.

### Mechanisms by which biguanides may protect against ischaemia/reperfusion injury

In ischaemic tissue lack of oxygen stops respiratory chain turnover and oxidative phosphorylation, and causes the mitochondrial NADH and succinate pools to become reduced. Under these conditions, the resting complex I converts into its deactive state [65], and (unless bound by the inhibitor protein IF_1_) ATP hydrolysis consumes ATP and contributes to maintaining the mitochondrial membrane potential [66]. Ischaemia/reperfusion injury occurs primarily upon reperfusion, when oxygen levels rise within the heavily reduced system and generate ROS; inhibition of complex I by rotenone is known to decrease ischaemia/reperfusion injury [67], perhaps because rotenone prevents superoxide production by reverse electron transport. The lack of reversibility of rotenone inhibition precludes this being a clinically relevant strategy, but recently the physiologically reversible complex I inhibitor, MitoSNO (mitochondria-targeted *S*-nitrosothiol), has been reported to protect ischaemic heart tissue from reperfusion-induced damage [68]. Furthermore, guanidinium-like unidirectional inhibitors of ATP hydrolysis have been reported to protect ischaemic rat heart myocardium [69], and metformin has been observed to decrease cardiac ischaemia/reperfusion injury in rodent models [6]. With an appropriate choice of biguanide it may be possible (subject to appropriate transport processes being present) to combine these effects on both complex I and ATPase into one molecule: biguanides bind to deactive complex I, inhibiting both forward catalysis (helping to control reactivation of the chain upon reperfusion) and superoxide production by reverse electron transport [70], and also inhibit ATP hydrolysis. Activation of AMPK in the ischaemic tissue may also contribute [71].

### Relevance to the anti-neoplastic effects of metformin

Pharmaco-epidemiological studies have suggested that metformin use is associated with decreased cancer risk and/or improved cancer prognosis [4,5]. Some of these studies have been questioned [72], but the anti-neoplastic activity of metformin has been clearly demonstrated in laboratory models [4,5,9]. Although the secondary insulin-lowering action of metformin may contribute in specific cases, interest in biguanides as potential anti-neoplastic agents relates to evidence that they induce energetic stress in cancer cells [4]. Functional and energized mitochondria are essential for the energy-intensive process of cell proliferation [73] and metformin-induced decreases in mitochondrial ATP synthesis may directly cause cell death in cancer cells intolerant of energetic stress, as well as have an anti-proliferative effect as the result of energy-conserving responses, mediated by the activation of AMPK and the inhibition of mTOR (mammalian target of rapamycin) [4]. Our results confirm that inhibition of NADH-linked respiration in cells and mitochondria by metformin stems from the direct inhibition of complex I catalysis, and highlight the importance of considering the biguanides as a molecular family. Each member combines the biguanide functional group with structural features that determine their pharmacokinetics, cellular distribution, and molecular interactions, and thus their clinical potency and specificity. In the future these properties may be exploited to inhibit oxidative phosphorylation in cancer cells which are particularly dependent on it, such as in prostate cancer [74], cancer cells with up-regulated OCT1 transporters, such in as ovarian cancer [61], or cancer cells with detrimental complex I mutations that render them hypersensitive to inhibitors [75]. Furthermore, enhanced understanding of the molecular pharmacology and mechanisms of biguanide–protein interactions may enable the design and investigation of new drugs of the biguanide class. Some of these may demonstrate improved efficacy for established applications such as in the treatment of diabetes, or be suitable for exploiting new therapeutic opportunities such as in oncology.
